# Hydrochlorothiazide use, sun exposure, and risk of keratinocyte cancer

**DOI:** 10.1186/s12889-022-13705-9

**Published:** 2022-07-02

**Authors:** Jeffrey J. VanWormer, Eseoghene B. Abokede, Richard L. Berg

**Affiliations:** 1 Center for Clinical Epidemiology and Population Health, Marshfield Clinic Research Institute, 1000 North Oak Ave, Marshfield, WI 54449 USA; 2 Allina Health, Minneapolis, USA; 3 Marshfield Clinic Research Institute, Marshfield, USA

**Keywords:** Hydrochlorothiazide, Sun, Skin Cancer, Prevention, Adults

## Abstract

**Background:**

Keratinocyte cancer (KC) rates are increasing in the U.S., particularly in older age groups. Use of hydrochlorothiazide (HCTZ), due to its photosensitizing properties, and high sun exposure are two known NMSC risk factors, but their synergistic effects are undetermined. The purpose of this study was to examine the development of NMSC between adults who did and did not use HCTZ, as well as those with high and low sun exposure.

**Methods:**

A retrospective case–control sample was assembled from adult patients in north-central Wisconsin (USA). Duration of HCTZ use and occupational sun exposure were extracted from electronic health records, along with a linked survey of lifetime sun exposure.

**Results:**

There were 333 cases and 666 controls in the analytical sample. A significant main effect was observed for HCTZ duration in the full sample. Under low sun exposure, the odds of NMSC was 14% greater for each additional year of HCTZ use (aOR = 1.14 [1.11, 1.18], *p* < 0.001). In a sensitivity analysis of participants age 70 years and over, there was a borderline significant (*p* = 0.086) HCTZ use by high sun exposure interaction, suggesting modestly increased HCTZ risk in older, high sun exposure adults.

**Conclusions:**

Consistent with prior studies, longer duration of HCTZ use was a predictor of NMSC in north-central Wisconsin adults. NMSC may be accelerated in HCTZ users with outdoor lifestyles, but future studies should attempt to further disaggregate specific effects of sun exposure time, HCTZ duration, and age on NMSC development.

## Background

High blood pressure is a major global health issue and among the leading preventable causes of premature death [1]. In combination with lifestyle modification and systems-level healthcare changes, antihypertensive medications are the mainstay therapy for blood pressure control [2, 3]. Hydrochlorothiazide (HCTZ) is among the most commonly prescribed antihypertensive agents [4], but it also results in well documented photosensitivity and phototoxicity [5] that can damage cell membranes and metabolic compounds related to cancer development [6]. Drug-induced photosensitivity increases the risk of several forms of cancer, most notably basal and squamous cell carcinomas [7, 8].

Exposure to ultraviolet (UV) radiation, usually by excessive time spent outdoors in the sun, is also a known risk factor for skin cancer development [9, 10]. For example, incident keratinocyte, or non-melanoma, skin cancer (KC) is higher in individuals who work in outdoor occupations with substantial UV exposure [11, 12]. Despite these well-established links between KC, sun exposure, and HCTZ use, there are few studies of whether sun exposure and HCTZ use may interact to accelerate KC risk. Studies on this topic have been limited by indirect markers of sun exposure such as the self-reported number of lifetime painful sunburns or residential address information indicative of living in sunnier regions. While some studies have adjusted for said markers of sun exposure in their examination of HCTZ use [13, 14], analyses of HCTZ effect modification by sun exposure are lacking, with just one known case–control study showing enhanced risk of squamous cell carcinoma among users of photosensitizing medications who also have a tendency to sunburn [15].

KC is the most common cancer worldwide, and rates for basal and squamous skin cell carcinomas have been increasing over the past several years, particularly in elderly subgroups [10, 16]. As such, control of modifiable KC risk factors is increasingly important. Both HCTZ use and sun exposure are associated with increased KC risk, but are also important for the management of high blood pressure control [2] and vitamin synthesis [17], respectively. Thus the identification of groups at greater KC risk could help balance/optimize medical decisions such as antihypertensive agent selection. Using a case–control design, the purpose of this study was to examine the development of KC between adults who did and did not use HCTZ, as well as those with high and low sun exposure.

## Methods

### Design

The source population included adult patients of the Marshfield Clinic Health System (MCHS). MCHS is an integrated healthcare system serving a large geographic region in north-central Wisconsin (USA) that is primarily composed of small towns and rural areas. The source population was limited to adult patients with reasonably complete capture of their medical care in MCHS data systems, which included members of MCHS’s Virtual Data Warehouse (VDW) population. The VDW is a medical research resource used as part of the broader Health Care Systems Research Network [18] that includes individuals who are members of the MCHS-affiliated health insurance plan (Security Health Plan of Wisconsin) and/or residents of the Marshfield Epidemiologic Study Area (MESA). Individuals in MESA reside in one of 24 postal codes in northern and central Wisconsin [19], with over 90% capture of all medical visits [20]. Adults who were VDW members for ≥ 1 year between 01/01/2000 and 12/31/2019 were eligible for this study. A nested case–control sample was assembled. As described further below, cases included 333 randomly selected patients with a confirmed first known KC during the study timeframe. For each case, two controls were randomly selected (risk set sampling) from KC-free patients and matched on gender, 2-year age increment, and a clinical encounter within two years of the index case’s KC date, as well as counter-matched on duration of HCTZ use. Two matched controls were selected from HCTZ categories counter to each case. Counter-matching is used at the point of sampling to increase the range of responses for a key exposure, in this case to ensure a breadth of long-term, medium-term, short-term, and non HCTZ users (described further below). This counter-matching procedure essentially improves the statistical efficiency for interaction analyses between HCTZ use and sun exposure [21, 22]. A precise, a priori sample size calculation was not performed given the lack of prior data on interaction analyses between HCTZ use and sun exposure. In addition, the sample size was limited due to resource constraints that could not support chart reviews and surveys on more than 1,000 participants.

### KC case status

Cases included those with a diagnosis of KC using International Classification of Disease (ICD) 9 and 10 codes indicative of basal, squamous, or unknown non-melanoma malignant neoplasm of dermatological origin (available upon request). Only cases with an ICD code for KC and corresponding positive biopsy result were included. Positive biopsies were confirmed using manual chart review. All KC cases were combined, as study funding constraints limited the available sample size, which precluded analyses in separate KC subtypes (e.g., squamous cell carcinomas or rarer subtypes). To avoid major confounders of KC associations, patients with a prior history of skin or other cancers, organ transplant, immunosuppressive therapy (e.g., azathioprine, cyclosporine), or human immunodeficiency virus were excluded from case case–control selection.

### Exposures

#### HCTZ use duration

Active HCTZ use was defined as those taking HCTZ as of their index enrollment encounter (KC date for cases, nearest clinical encounter for matched controls) and with at least one prior prescription for HCTZ or HCTZ combination agents for six continuous months prior to the index date. The total duration of HCTZ was defined as the number of years of HCTZ use prior to the index date. As described previously, for the purposes of counter-matching (to ensure a breadth of HCTZ users for interaction analyses), HCTZ use was divided into 3 groups based on observed frequencies: none, < 6 years (short- or medium-term use), and ≥ 6 years (long-term use). For analyses, HCTZ was modeled as the actual duration of use in years.

#### Sun exposure

**Fig. 1:**
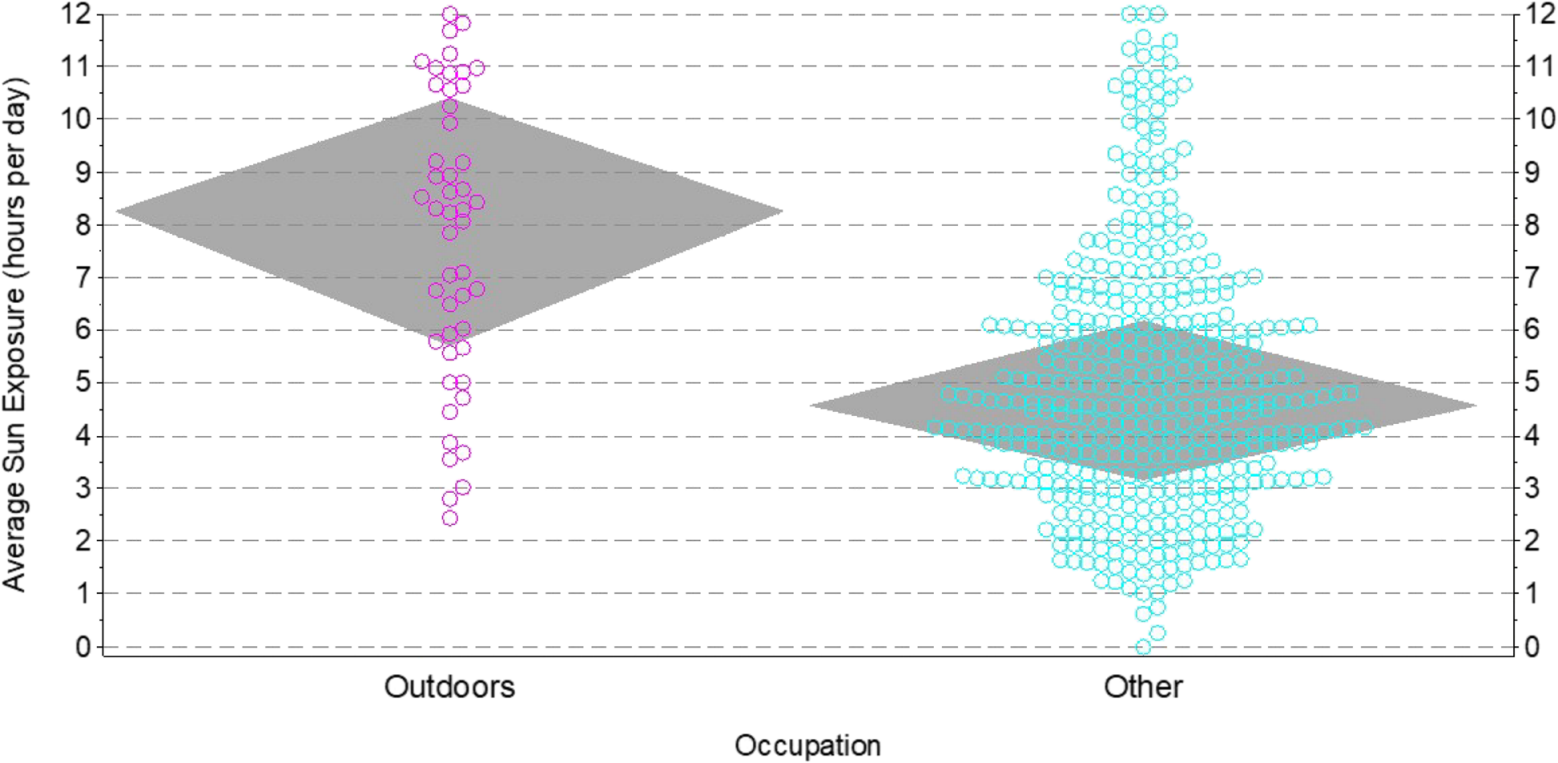
Lifetime daily hours of sun exposure (shaded regions show medians and interquartile ranges) by occupation in north-central Wisconsin adults

Sun exposure was assessed using two methods that were later combined, including electronic health records (EHR) review of high sun exposure occupational history and survey of self-reported time spent in the sun across the lifespan. Specifically, occupational sun exposure was first collected by chart review of all patients and their documented occupation (within 10 years prior to their index date) indicative of regular sun exposure. Informed by prior meta-analyses [11, 12], this entailed identifying patients who worked in outdoor occupations such as agriculture, construction, mail carrier, and similar positions. Those without a documented employment history or who worked mainly indoors were (initially) considered as lower occupational sun exposure. Presumably due to their older average age, EHR-based occupation could only be found on 53% of the sample. Thus to provide additional information on sun exposure, all available cases and controls were also solicited to complete a brief survey on lifetime sun exposure (that included an initial mailed invitation letter, along with up to four phone follow-up contacts to encourage responding). Items from the survey developed in the U.S. Radiologic Technologists Study were used [23, 24]. Respondents reported time spent outdoors across ~ 20-year age ranges throughout their lifespan. Based on our internal validation testing of the correlations between high occupational sun exposure and self-reported lifetime sun exposure, participants who reported a mean of ≥ 6 h per day of sun exposure over their lifetime appeared to show the clearest ‘break’ point in that the strength of said correlation diminished at higher levels (see Fig. 1). Thus for analytical purposes, we operationally defined participants with either ≥ 6 h per day of mean lifetime sun exposure, or a high sun exposure occupation from the EHR, as high sun exposure. All other participants were categorized as lower sun exposure, which we refer to as simply ‘low’ sun exposure hereafter to improve interpretability.

#### Covariates

Several covariates were also extracted from EHR data, including the age, gender, body mass index, and Charlson comorbidity score [25] closest to each participant’s index date. All study procedures were approved in advance by the Marshfield Clinic Institutional Review Board, with a request to waive documentation of informed consent. The study was also conducted in accordance with the Declaration of Helsinki.

### Analyses

Descriptive statistics were used to characterize KC cases and controls. In the multivariable modeling, weighted conditional logistic regression was used to determine main and interactive associations between KC case status, HCTZ duration, and sun exposure. As outlined in more detail by Cologne and colleagues [26], this is a standard analytical approach in counter-matched designs, with sampling weights used as offsets, added to the regression model to account for the weighted selection. In addition, models were adjusted for age, Charlson comorbidity score, and years of data capture prior to index date. Given the heavy influence of age on KC [10, 16], age was adjusted for as a linear and splined covariate. In addition, a planned subgroup analysis was conducted that was restricted to the subsample of participants age 70 years and older.

## Results

There were 999 individuals in the analytical sample, including 333 KC cases plus 666 KC-free controls. Descriptive characteristics of cases and controls are outlined in Table 1. KC cases (and thus matched controls) had a mean age of 71 years and 51% were female. The mean number of years on HCTZ prior to index date was 4.9 years among cases. Sun exposure surveys were mailed to 846 study participants who were still living and with a current address in MCHS data systems. The survey response rate was 56% (*n* = 473). With the exception of age (respondents were, on average, 1.9 ± 12.2 years older, *p* = 0.027), respondents and non-respondents were similar on all descriptive characteristics. The proportion of participants operationally defined as having high sun exposure was just slightly more common in cases relative to controls (24% vs. 20%, *p* = 0.192), and the majority of adults with high sun exposure were male (79%).

**Table 1:** Baseline characteristics of adults with and without non-melanoma skin cancer in north-central Wisconsin

	KC cases^a^ *n* = 333	KC-free controls *n* = 666	*p*
Mean age (years)	71.7 ± 13.7	71.6 ± 13.5	0.996
Gender			
Female	170 (51%)	340 (51%)	1.000
Male	163 (49%)	326 (49%)	
Race/ethnicity			
White, Non-Hispanic	324 (97%)	649 (97%)	0.453
Not White or Hispanic	2 (1%)	8 (1%)	
Unknown	7 (2%)	9 (1%)	
Charlson comorbidity score (points)	1.1 ± 1.6	0.9 ± 1.5	0.346
Body mass index (kg/m^2^)	31.0 ± 6.7	31.3 ± 6.8	0.442
Time in cohort before index date (years)	13.3 ± 8.0	12.7 ± 7.5	0.170

*KC*  Keratinocyte cancer

^a^Values are reported as frequency (% of column total) or mean ± SD

**Fig. 2:**
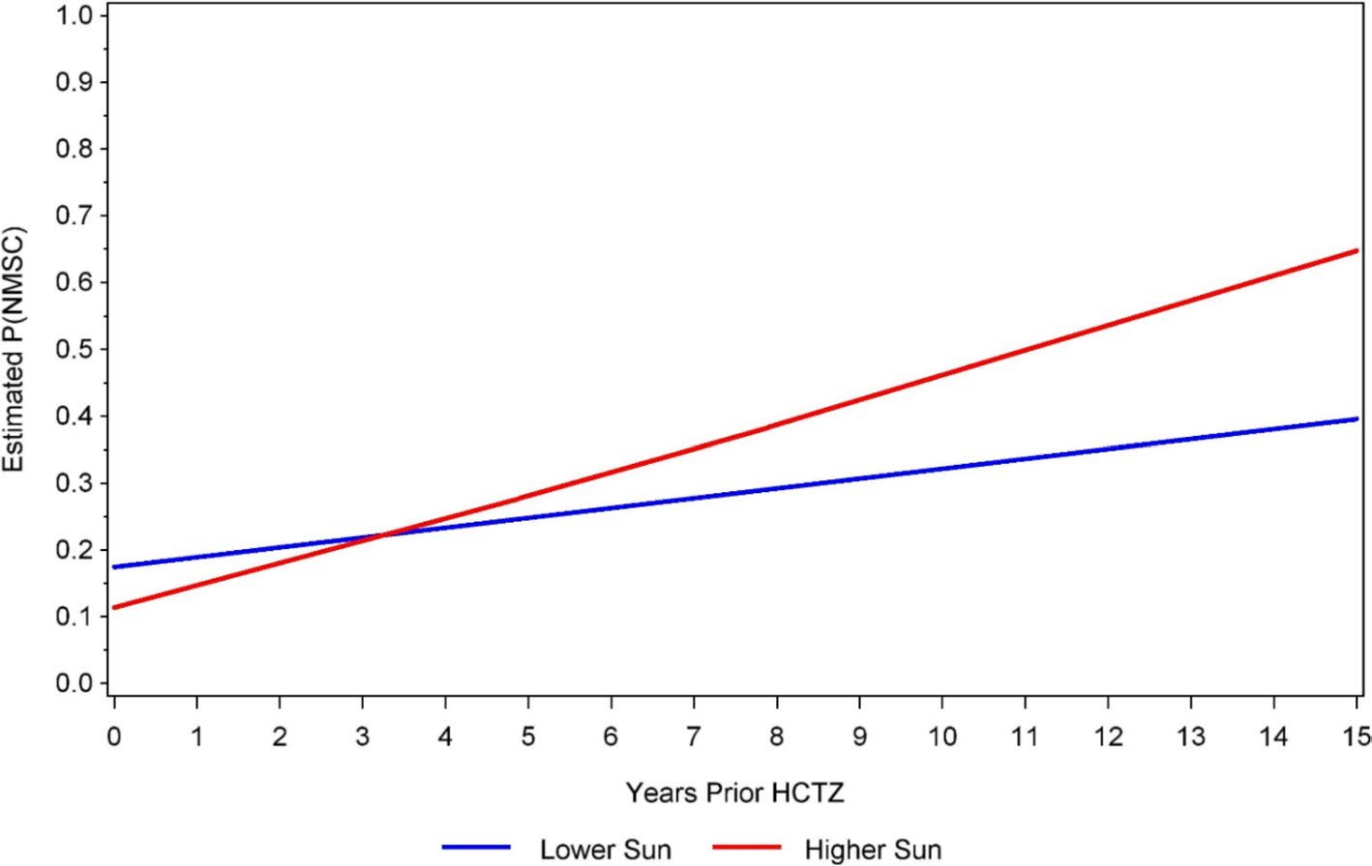
Model-estimated probability (P) of keratinocyte cancer (KC) by duration of hydrochlorothiazide (HCTZ) use and high vs. low sun exposure in north-central Wisconsin adults age 70 and older (*n* = 434)

As outlined in Table 2, HCTZ duration showed a significant main effect in the final interaction model. Under low sun exposure, the odds of KC was 14% greater for each additional year of HCTZ use (aOR = 1.14 [95% CI: 1.11, 1.18], *p* < 0.001). High sun exposure, as well as the HCTZ duration by high sun exposure interaction, showed weaker, non-significant associations with KC. Charlson comorbidity score and age (splined) were significant covariates. Given the variability of KC risk by age, a planned sensitivity analysis followed that was restricted to the 434 adults in the sample who were age 70 years and over. The main effect for HCTZ duration in this older age strata was again a significant predictor of KC (aOR = 1.08 [95% CI: 1.03, 1.12], *p* = 0.001) and the HCTZ use by high sun exposure interaction was borderline significant (*p* = 0.086). To better illustrate this association, the predicted probability of KC by HCTZ duration and sun exposure groups is described in Fig. 2.

**Table 2:** Multivariable logistic regression model of the association between keratinocyte cancer, hydrochlorothiazide duration, and sun exposure, along with covariates, in north-central Wisconsin adults (*N* = 999)

	Keratinocyte Cancer^a^
Age (linear years)	0.60 (0.33, 1.09), *p* = 0.091
Age (spline)	1.96 (1.11, 3.47), *p* = 0.020
Charlson comorbidity score (points)	1.18 (1.06, 1.32), *p* = 0.003
Time in cohort before index date (years)	1.00 (0.98, 1.03), *p* = 0.689
Hydrochlorothiazide duration (years)	1.14 (1.11, 1.18), *p* < 0.001
Sun exposure	
High	1.16 (0.71, 1.48), *p* = 0.563
Low	–- ref –-
Interaction	1.02 (0.97, 1.09), *p* = 0.643
Hydrochlorothiazide duration × High sun exposure	1.16 (1.10, 1.23)
Hydrochlorothiazide duration × Low sun exposure	1.14 (1.11, 1.18)

^a^Values are reported as odds ratio (95% confidence interval) and p-value of keratinocyte cancer, relative to the reference category for categorical exposures or a 1-unit increase for continuous exposures

## Discussion

To our knowledge, this was the first study to examine the interaction between HCTZ use and individual-level lifetime sun exposure. Consistent with prior studies [27], HCTZ was associated with KC in north-central Wisconsin adults, as each additional year of HCTZ use correlated with a 14% increase in KC risk. The degree to which HCTZ interacted with high sun exposure to further increase KC risk, however, was generally minimal. High lifetime sun exposure in combination with HCTZ duration was not significantly associated with KC in the full sample, but there was a statistically borderline indication of increased KC risk in HCTZ users with high sun exposure in the subset of participants age 70 years or older. This increased risk of HCTZ use in older, high sun exposure adults was rather modest though, perhaps most noticeable in those with over a decade of prior HCTZ use.

Concerns about HCTZ and sun interactions are well founded. High UV exposure has long been known to increase the risk of severe sunburn and skin cancer [9, 10] and, in response to recent evidence from very large observational studies [14, 28, 29], HCTZ labeling in the U.S. now has overt warnings of increased KC risks [30]. KC is fast becoming a disease (primarily) of the elderly [10, 31], likely reflecting accrued exposures to KC risk factors (e.g., sun seeking, ozone depletion) over longer lifetimes [32, 33]. For example, a recently retired 70 year old farmer presenting with KC may have accumulated considerable time on HCTZ while they were also still working long days in the sun. This may not (yet) be the case in younger individuals, even with an otherwise similar risk factor profile. More research is needed in larger prospective samples to disaggregate specific effects of sun exposure time, HCTZ duration, and age of KC development and clinical presentation.

This study was strengthened by the sampling of cases and controls from a defined population with reasonably complete capture of prior medical care/history within an integrated healthcare system. The chief limitation was the observational design, including unmeasured confounding and measurement errors, that did not permit causal conclusions. Study funding constraints did not permit assembly of a larger sample due to the need for chart review, which limited statistical power and prohibited analyses by KC subtype, despite the counter-matching method. It appears most prior research has predominantly observed (separate) associations between squamous cell carcinoma and HCTZ use and sun exposure, thus it is important for future studies to use larger samples that can examine effects in specific KC subtypes. If, for example, squamous cell carcinoma is more reactive to sun exposure than basal cell carcinoma, the combination of all KC subtypes as a composite outcome could mask more meaningful interactions between sun exposure, HCTZ use, and squamous cell carcinoma (resulting in a false negative finding). Sun exposure misclassification was also a concern, as this measure was rather blunt and required the combination of both occupational history extracted from the EHR and survey based recall of hours spent in the sun over one’s lifetime. These are indirect, estimated measures of total UV ‘dose’ and some participants were likely incorrectly classified as low sun exposure due to limited occupational data in the EHR, or survey non-response or recall bias. In addition, the timing of our sun exposure metric was broad; applicable to a given participant’s lifetime and/or career. It was not possible to tie a finer grain measure of sun exposure directly to the timeframe of HCTZ use, which may be a more specific catalyst of KC risk worth exploring in future studies. Though some major photosensitizing medications were controlled for in the exclusion criteria, all possible photosensitizing medications were not adjusted for in our analyses. Finally. generalizability was also limited by the predominantly rural, racially homogenous source population.

## Conclusions

KC is a rising public health risk, which underscores the need for better control of KC risk factors. Consistent with prior studies [27], longer duration of HCTZ use was a predictor of KC in this study of north-central Wisconsin adults. This risk of KC may also be somewhat accelerated in older HCTZ users with high sun exposure. If confirmed in other prospective studies, our findings may have implications for the selection of optimal antihypertensive medications and the importance of minimizing UV exposure (e.g., protective clothing), particularly given broader trends toward an aging population with greater high blood pressure and KC needs.

## Data Availability

The data that support the study conclusions are unavailable for public access because informed consent to share said data (beyond the research team) was not obtained from study participants, but are available from the corresponding author on reasonable request.

## Abbreviations

USA: United States of America
KC: Keratinocyte cancer
HCTZ: Hydrochlorothiazide
UV: Ultraviolet
MCHS: Marshfield Clinic Health System
VDW: Virtual Data Warehouse
MESA: Marshfield Epidemiologic Study Area
ICD: International Classification of Disease
EHR: Electronic health records
aOR: Adjusted odds ratio
CI: Confidence interval
